# “Chatting” about DISE: can machine learning tools assist decision making for obstructive sleep apnea?

**DOI:** 10.1093/sleep/zsag118

**Published:** 2026-05-11

**Authors:** Ofer Jacobowitz

**Affiliations:** ENT and Allergy Associates, New York, NY, United States; Zucker School of Medicine, Hofstra University, Hempstead, NY, United States

In the quest for personalized treatment for obstructive sleep apnea (OSA), methods for phenotyping and patient selection are essential. Drug-induced sleep endoscopy (DISE) is a current method for dynamic upper airway evaluation for potential sites for treatment. DISE complements other evaluation methods including awake endoscopy, tongue position, and radiographic imaging. In the USA, DISE is now commonly used due to its requirement in selecting patients for certain implantable hypoglossal neurostimulation systems, while in Europe it has been used for decades for surgical and mandibular advancement device selection. Although DISE appears advantageous compared with assessments while awake, it is subject to inter-rater variability and other limitations [1].

The variability of DISE extends well beyond differences in how clinicians score the exam. Pharmacologically-induced sleep will vary depending on drug administration method, pharmacokinetic, and pharmacodynamic properties [2]. Likewise, DISE is not true sleep, and its brief duration will not replicate the variability of OSA across an entire night’s sleep [3]. DISE can vary in terms of positioning of the endoscope tip, difficulty of visualization due to pharyngeal collapse or saliva, a patient’s susceptibility to sedation and reflexes. Additionally, DISE only provides a view of the airway lumen irrespective of maxillary or mandibular hypoplasia or tissue composition. Given these limitations, there is interest in use of machine-learning approach for standardization of analysis and novel insights.

In the study by Hack and colleagues [4], the ChatGPT-4.0 artificial intelligence (AI) tool was used to try to standardize analysis of DISE videos and make treatment recommendations in the context of clinical data. An analysis of two separate runs of the AI tool for 16 patient videos was compared to that of two expert otolaryngologists, a resident, and the operative surgeon. There was very high concordance for the V-O-T-E (Velum-Oropharynx-Tongue-Epiglottis) collapse levels and degree of collapse except at the epiglottis. Using the video analysis in conjunction with clinical data that included some symptoms, comorbidities and prior treatments, the AI tool made selections from a specified list of recommendations for appropriate treatment, mostly in agreement with the medical specialists. There were four discordant recommendations for treatments by GPT-4.0 compared to that of the reference surgeon, but none were inappropriate nor aggressive.

The study is preliminary and is limited by including a small number of patients from a single center with various OSA severities and incomplete clinical data sets. DISE videos were ten minutes in duration though collapse pattern may change with longer observation [3]. The full physiological context of DISE was not captured by the videos alone. The reference standard for the VOTE score and treatment plan was that of the operative surgeon which is subject to personal bias. Likewise, agreement between GPT-4.0 and four surgeons from the same center is not sufficient to establish universal agreement. However, the potential for use of AI suggested in this paper is very interesting, to hopefully reduce variability of DISE assessment in the field and occasionally suggest overlooked findings or options for treatment.

There is clinical equipoise whether DISE can improve outcomes for upper airway surgery. In retrospective and prospective studies for HGNS, and upper airway surgery, some DISE patterns correlate with outcomes but studies have small samples. For a distal HGNS system, absence of Complete Circumferential Collapse (CCC) of the velopharynx was identified as a key predictor of Apnea Hypopnea Index (AHI) reduction, ultimately leading to CCC exclusion requirement for distal HGNS qualification, yet it was only based on five patients with CCC [5]. Subsequently, in retrospective studies, absence of complete Lateral Wall collapse (LW) was identified as the component of CCC predictive of worse outcome. In one multicenter study, absence of LW increased the AHI response from 58% to 74% [6, 7], albeit in a LW sample of 19. Similarly, in a single center prospective study, 27 patients with LW had an AHI success of 32% compared with 63% in absence of LW, and corresponding AHI reductions of 15 and 23 events/hour [8]. It is worth noting that the relevance of DISE may not universally apply to all HGNS systems as a proximal HGNS system does not require DISE for selection [9, 10].

For upper airway surgery, the data is mixed for the utility of DISE-guided surgery, as noted in a recent systematic review, with an overall additional 9-event/hour AHI benefit and additional 3-point Epworth Sleepiness Score drop over non-DISE guided surgery [11]. In several retrospective and prospective, single- and multicenter studies, LW and CCC were associated with lesser AHI reduction and lower success across a range of upper airway surgeries [12–14]. To control heterogeneity, a randomized controlled trial was conducted of barbed reposition pharyngoplasty *alone* with or without DISE preoperatively in 110 patients [15]. In the DISE arm, patients with total tongue base or epiglottic collapse were excluded with resultant additional AHI reduction of 7 events/hour and higher success rate of 83% vs 60%. What remains unknown is how the excluded patients would have fared with pharyngoplasty. Other studies cast doubt on utility of outcomes with tongue collapse during DISE. In a single center study of 38 patients, complete tongue base collapse or its absence did not affect outcome of *palatopharyngoplasty alone* with reduction of AHI from mean 45 events/hour to about 15 events/hour in both groups [14]. Likewise, in prospective multicenter studies of multilevel surgery, and in a randomized trial in which DISE was compared with awake examination to guide multilevel surgery for severe OSA, no differences in AHI or oximetry outcomes were observed between the groups [16, 17]. In another study, treatment or *non-treatment* of DISE collapse sites did affect the AHI outcome [18]. The discrepancies could be explained in part by unconscious bias in patient selection, procedure selection or performance. It is also possible that treating upstream collapse using palatopharyngoplasty reduces downstream collapse at the Tongue base level or epiglottis for some patients [14], as was simulated by in a study using a nasopharyngeal tube in DISE [19].

For decades DISE has been performed only using head or body positioning, jaw advancement maneuvers, and rudimentary assessment of airflow. The utility of DISE for outcomes can likely be strengthened by incorporation of maneuvers, additional sensors, and analyses. For upper airway surgery, assessment of stabilization of oximetry while applying anterior traction to the palate using DISE was predictive of surgical response to palatopharyngoplasty in a small but severe OSA cohort [20]. Likewise, the addition of cardiorespiratory polygraphy monitoring during DISE with jaw repositioning was highly predictive of oral appliance AHI outcome in one study [21]. More recently, investigators have integrated positive airway pressure in DISE, correlating flow changes during stepwise pressure reduction with pharyngeal imaging and morphology [22, 23]. These airflow measurements may add a critical and missing dimension for DISE. Finally, a broader reconsideration of how we interpret DISE videos is timely. Focusing on the terminal collapse at sites may not reveal the therapeutic target. Perhaps AI can provide us a “software update” for novel analysis of DISE image patterns, provided that we reboot DISE with additional signals. At the same time, clinical decision making for OSA management remains complex and any treatment recommendations generated by AI must be approached with considerable caution.
